# Hybrid Origins of *Carex rostrata* var. *borealis* and *C*. *stenolepis*, Two Problematic Taxa in *Carex* Section *Vesicariae* (Cyperaceae)

**DOI:** 10.1371/journal.pone.0165430

**Published:** 2016-10-25

**Authors:** A. Tiril M. Pedersen, Michael D. Nowak, Anne K. Brysting, Reidar Elven, Charlotte S. Bjorå

**Affiliations:** 1 Natural History Museum, University of Oslo, Oslo, Norway; 2 Centre for Ecological and Evolutionary Synthesis (CEES), Department of Biosciences, University of Oslo, Oslo, Norway; Leibniz-Institute of Freshwater Ecology and Inland Fisheries, GERMANY

## Abstract

Hybridization is frequent in the large and ecologically significant genus *Carex* (Cyperaceae). In four important sections of the northern regions (*Ceratocystis*, *Glareosae*, *Phacocystis* and *Vesicariae*), the frequent occurrence of hybrids often renders the identification of “pure” species and hybrids difficult. In this study we address the origins and taxonomic rank of two taxa of section *Vesicariae*: *Carex rostrata* var. *borealis* and *C*. *stenolepis*. The origin and taxonomic status of *C*. *stenolepis* has been the subject of substantial debate over the years, whereas *C*. *rostrata* var. *borealis* has received very little attention in the years since its first description in the 19^th^ century. By performing an extensive sampling of relevant taxa from a broad distribution range, and analyzing data from fifteen microsatellite loci developed specifically for our study together with pollen stainability measures, we resolve the hybrid origins of *C*. *rostrata* var. *borealis* and *C*. *stenolepis* and provide new insights into this taxonomically challenging group of sedges. Our results are in accordance with previous findings suggesting that *C*. *stenolepis* is a hybrid between *C*. *vesicaria* and *C*. *saxatilis*. They are also in accordance with a previous proposition that *C*. *rostrata* var. *borealis* is a hybrid between *C*. *rostrata* and *C*. *rotundata*, and furthermore suggest that both hybrids are the result of multiple, recent (i.e., postglacial) hybridization events. We found little evidence for successful sexual reproduction within *C*. *rostrata* var. *borealis* and *C*. *stenolepis*, but conclude that the common and recurrent, largely predictable occurrence of these taxa justifies accepting both hybrids as hybrid species with binomial names. There are, however, complications as to types and priority names, and we therefore choose to address these problems in a separate paper.

## Introduction

Hybridization is common among vascular plants, and is especially frequent in the large genus *Carex* (Cyperaceae). As an example, among the more than one hundred *Carex* species present in Norway, approximately 60% partake in some degree of hybridization, producing 113 hybrid combinations [1]. A majority of these hybrids are between species in four sections: *Ceratocystis*, *Glareosae*, *Phacocystis*, and *Vesicariae*. This phenomenon is common and particularly frequent in the same sections in other geographic regions as well (e.g., [2, 3]). With its approximately 2000 species, the genus *Carex* is among the largest genera of vascular plants [3–5]. It is also one of the most widespread and ecologically significant of plant genera, occupying a multitude of habitats on all continents except Antarctica [2, 4–7]. The majority of *Carex* species are found in cold and temperate regions in the northern hemisphere [2]. In arctic habitats, they are often dominant and abundant both in terms of species richness and biomass, and thus also important in characterizing different vegetation types [8] (see also references in [7]).

Despite their ecological importance, *Carex* species are often ignored in practical fieldwork due to their complex taxonomy and sometimes less distinctive characters, which complicates species identification [7, 9]. The frequent occurrence of hybrids, and of partly or fully fertile hybrids in certain sections, is furthermore suspected to compromise the taxonomic status of many species [2, 9–12] (see also [13]). While a number of recent morphological and molecular studies (several listed in [11]) have helped clarify the phylogenetic relationships of many groups and species of *Carex*, the genus is nevertheless in need of a modern worldwide revision. There are, however, modern treatments available for two of the major parts of the Northern Hemisphere: the previous Soviet Union [2] and North America including Greenland [3], and a global phylogenetic hypothesis of the genus was recently published by the Global *Carex* Group [14].

*Carex* section *Vesicariae* (Heuff.) J. Carey is one of the sections with abundant hybridization and several potentially fertile and semi-fertile hybrid taxa [1, 3, 10, 15, 16]. The section encompasses 30 – 45 species worldwide [2, 17]. Following Egorova’s treatment [2], at least six species are found in northern Europe: *Carex rhynchophysa* C.A. Mey., *C*. *rostrata* Stokes, *C*. *rotundata* Wahlenb., *C*. *saxatilis* L., *C*. *stenolepis* Less., and *C*. *vesicaria* L., perhaps also *C*. *pseudocyperus* L. (see [17]). Elven et al. [16] furthermore included an additional taxon, *C*. *rostrata* var. *borealis* (Hartm.) Kük., in their treatment of the section, and reported two subspecies for *C*. *saxatilis* (subsp. *saxatilis* and subsp. *laxa* (Trautv.) Kalela). Whether *C*. *saxatilis* should be divided in this way is, however, disputed (see e.g., [16–19]).

The majority of the species of *Vesicariae* grow in swamps, mires, along water courses and in shallow water. Together with a few species of some smaller sections, and the species of section *Phacocystis* Dumort., they are among the most important constituents of boreal and arctic wetlands, dominating in many vegetation types due to their growth habit [8, 20]. They are all rhizomatous with sympodial growth [21], where the main underground shoots are horizontal and end in vertical leafy shoots and culms, whereas branches at the base of the leafy shoots continue the horizontal growth. The length of the rhizome between aerial shoots determines whether the plants form more or less open mats or tussocks; all North European plants tend to be mat-forming rather than tussock-forming.

*Carex stenolepis* and *C*. *rostrata* var. *borealis* are intermediate between their assumed parents in growth features, most often with denser stands than *C*. *saxatilis* s. str. and *C*. *rotundata*. The horizontal rhizomatous growth in all of the above plants results in homogenous stands, which are often very large. An extensive species stand with thousands of separate aerial shoots may therefore consist of a single genetic clone, or possibly very few clones. The life span of such clones is unknown, but the postglacial history of the northern European mires suggests that they may occupy the same area for hundreds or perhaps thousands of years. However, it is unlikely that such clones have remained in place throughout the entire postglacial period (i.e., the last 10 ‒ 15 thousand years), as the extent and position of mires has changed over time (as proposed by Blytt [22]).

*Carex rostrata*, *C*. *rotundata*, *C*. *vesicaria* and *C*. *saxatilis* are distinguished from each other by several reliable morphological characters (e.g., [2]), but the four species are closely related and thought to hybridize freely wherever their ranges overlap. Primary hybrids from all six possible combinations of these species have been reported [1, 17, 23]. In addition to intrasectional hybrids, hybrids with species from sections *Carex*, *Lupulinae*, *Paludosae*, *Pseudocypereae* and *Tumidae* have also been observed [1, 16, 23–25]. For the most part, both intra- and intersectional hybrids seem to be nearly or fully sterile, but possibly fertile exceptions exist (e.g., [23]).

The taxa *C*. *rostrata* var. *borealis* and *C*. *stenolepis* may represent fertile hybrids within section *Vesicariae*. Both taxa seem to combine characters from universally accepted species within their section; *C*. *rostrata* var. *borealis* combines characters from *C*. *rostrata* and *C*. *rotundata* [16], whereas *C*. *stenolepis* combines characters from *C*. *vesicaria* and *C*. *saxatilis* (see e.g., [24, 26]). In contrast to the pollen-sterile primary hybrids between *C*. *rostrata* and *C*. *rotundata*, Elven [27] stated that both anthers and pollen are usually well developed in *C*. *rostrata* var. *borealis*. Because of this, he assumed *C*. *rostrata* var. *borealis* to be a possibly sexually reproducing species of hybrid origin resulting from crosses between *C*. *rostrata* and *C*. *rotundata*, presumably having restored some of its fertility through back-crossing with the latter. Field observations by A.T.M. Pedersen and R. Elven (pers. obs.) support this view. As *C*. *rostrata* var. *borealis* was regularly found to behave as an independent entity, often growing in mixed stands with only one of the presumed parental species, Elven [27] proposed “that var. *borealis* may be either a homogenized backcross product towards *C*. *rotundata*, regaining fertility and acceptable as a hybrid species, or an entirely independent species”. Half a century earlier, Drury [15] described *C*. *paludivagans* from the upper Kuskokwim River region of Alaska, a taxon he too considered to be a stabilized, fertile hybrid between *C*. *rostrata* and *C*. *rotundata*. Studies by Ford et al. [28], however, showed that Drury’s “*C*. *rostrata*” specimens were in fact *C*. *utriculata*, and *C*. *paludivagans* has since been regarded as the offspring of *C*. *rotundata* and *C*. *utriculata*. According to Egorova [2], *C*. *utriculata* does not occur in Eurasia (see [16]).

The taxonomic status of *Carex stenolepis* has long been disputed. Jakobsen [29] found it to be morphologically distinct, and accepted it as a species. Hylander [24] too had previously listed several arguments why *C*. *stenolepis* might be treated at species level. P.W. Ball in Elven et al. [16] was very critical to the taxon and claimed that it simply represented primary hybrids between *C*. *saxatilis* and *C*. *vesicaria*, as he had only found empty perigynia in the plants. T.V. Egorova seemed to be of the same opinion [16], despite having previously treated *C*. *stenolepis* at species level [2]. Elven [27] wrote that unlike pollen production, failure of fruit production in sections *Vesicariae* and *Carex* does not necessarily point to hybridity. Fruit production often fails even in established species such as *C*. *rotundata* and *C*. *rostrata* possibly due to self-incompatibility in large clones of these highly rhizomatous species, or due to unfavourable climatic conditions during flowering (see, e.g., [24], concerning *C*. *stenolepis*). Like *C*. *rostrata* var. *borealis*, plants of *C*. *stenolepis* often seem to have well developed anthers and pollen grains, and both taxa are capable of forming large and morphologically uniform stands, often in the absence of one or both of their putative parental species [16, 24, 26, 28]. Furthermore, both taxa are not exactly morphologically intermediate between their putative parents, but rather seem closer to one parent (*C*. *rostrata* var. *borealis* is closer to *C*. *rotundata* and *C*. *stenolepis* closer to *C*. *saxatilis*; field observations by A.T.M. Pedersen and R. Elven, [27]), possibly indicating back-crossing between primary hybrids and one of their parents.

In this study we apply genetic data to address the taxonomic status of *C*. *rostrata* var. *borealis* and *C*. *stenolepis*. More specifically, we address the following questions: 1) Is *C*. *rostrata* var. *borealis* an interspecific hybrid between *C*. *rostrata* and *C*. *rotundata*? 2) Is *C*. *stenolepis* an interspecific hybrid between *C*. *vesicaria* and *C*. *saxatilis*? 3) If one or both of these taxa appear to be the product of hybridization, can these data help us to determine if these hybrid lineages formed once or multiple times? 4) Are *C*. *rostrata* var. *borealis* and *C*. *stenolepis* capable of sexual reproduction and might they thus potentially be (partly) sexual species reproducing by seeds, more or less independent of their parents? In order to answer these questions we examined genetic differentiation among several populations of the putative hybrids and their respective parental species at 15 microsatellite loci, and measured pollen stainability in *C*. *rostrata* and *C*. *rotundata* and their putative hybrid *C*. *rostrata* var. *borealis*. Similar investigation of *C*. *stenolepis* and its putative parents could not be performed due to lack of available plants of *C*. *stenolepis* in optimal flowering stage among the collected material.

## Materials and Methods

### Sampling and DNA extraction

In this study we included a total of 193 samples from 121 sample sites of the taxa *C*. *rostrata* (72 samples), *C*. *rostrata* var. *borealis* (26), *C*. *rotundata* (28), *C*. *vesicaria* (25), *C*. *stenolepis* (14) and *C*. *saxatilis* (28 samples, mainly subsp. *saxatilis* but including some samples assigned in the field to subsp. *laxa*). Sampling took place during the summers of 2012 and 2013 and was carried out over a broad geographic area in an attempt to match the distribution range of the different taxa in Fennoscandia (and Iceland) in the best way possible (S1 Table, S1 Fig). The taxa included in this study are all common and not protected, and no specific sampling permits are required outside protected areas in the Nordic countries. Whenever possible, five shoots of a taxon were collected from each sample site. Due to the highly rhizomatous growth form of the plants of section *Vesicariae*, and in an attempt to avoid sampling only within clones, shoots were ideally sampled at a 5 – 10 m distance or more from each other. In addition to the field samples, two Swiss samples of *C*. *rostrata* were accessed from the herbarium of Oslo (O).

A small amount of fresh leaf material from each shoot was dried in silica gel for subsequent DNA extraction and microsatellite analyses, while the shoot itself was pressed and used as voucher specimen. The dried leaf samples and their associated vouchers are deposited at the herbarium of the Natural History Museum, University of Oslo (O). Prior to DNA isolation, approximately 10 mg dry leaf tissue per sample was ground with tungsten carbide beads (Qiagen, Hilden, Germany) for 1 – 1.5 min x 2 in a Retsch MM301 mixer mill (Haan, Germany). DNA was extracted using the DNeasy Plant Mini Kit or DNeasy 96 Plant Kit (Qiagen) following the manufacturer’s protocol with the exception that elution was performed using 75 μL AE buffer instead of 50 μL.

The samples included in this study were divided into three different datasets; one named the *C*. *rostrata* var. *borealis* dataset, containing all samples of *C*. *rostrata* var. *borealis* and its putative parental species *C*. *rostrata* and *C*. *rotundata*, one named the *C*. *stenolepis* dataset, containing all samples of *C*. *stenolepis* and its putative parental species *C*. *vesicaria* and *C*. *saxatilis*, as well as a dataset named the total dataset, which included all samples from the former two datasets combined.

### Microsatellite development and analysis

Three leaf samples from each of the four putative parental species (*C*. *rostrata*, *C*. *rotundata*, *C*. *vesicaria* and *C*. *saxatilis*) were sent to ecogenics GmbH (Zürich, Switzerland) for the development of diagnostic microsatellite loci capable of distinguishing between the species. An SSR-enrichment protocol using magnetic steptavidin beads and biotin-labeled CT and GT repeat nucleotides was performed, and the enriched library was sequenced on an Illumina MiSeq platform using the Nano 2x250 v2 format. A total of 8404 contigs and singlets were produced after assembly, of which 1361 were found to contain a microsatellite insert. Of these possible microsatellite candidates, 776 were suitable for primer design. From the total of 36 primer pairs that had been tested for polymorphism in the 12 *Carex* samples and ultimately delivered from ecogenics, we selected 22 primer pairs that showed most potential for discriminating between the putative parental species, and these were accommodated into five multiplex groups using Multiplex Manager [30]. Fifteen microsatellite loci were found to consistently amplify in our target species and were thus applied for all further analyses. Fluorescently labelled forward primers with FAM, HEX or ATTO 550 were ordered from Eurofins (Ebersberg, Germany).

Each multiplex PCR was performed using Type-it Microsatellite PCR Kit (Qiagen) with a final volume of 10 μL containing 5 μL Type-it Master Mix, 2 μL RNAse-free water, 1 μL primer mix (0.2 μM of each primer) and 2 μL 10X diluted template DNA. All PCR amplifications were conducted using a T100 Thermal Cycler (Bio-Rad Laboratories, Inc.) under the following conditions: initial denaturation at 95°C for 5 min followed by 30 cycles of 95°C for 30 s, 57°C for 90 s, and 72°C for 30s, with a final extension at 60°C for 30 min. A mixture of 1 μL 1:30 diluted PCR product, 8.85 μL HiDi formamide and 0.15 μL GeneScan ROX 500 size standard (Applied Biosystems, Warrington, UK) was denatured at 95°C for 5 min before fragments were separated by capillary electrophoresis using an ABI 3130*xl* Genetic Analyzer (Applied Biosystems, Foster City, CA, USA). All 96-well plates contained negative controls and replicates in addition to the multiplex reactions.

### Data analysis

Alleles were scored using Geneious (v. 6.1.8, Biomatters Ltd., [31]). Genetic differentiation among the different *Carex* taxa was visualized using principal components analysis (PCA) in NTSYSpc version 2.11a [32] separately on the *C*. *rostrata* var. *borealis* and *C*. *stenolepis* datasets. Bayesian cluster analyses were performed in STRUCTURE v. 2.3.3 [33] using the Lifeportal service at the University of Oslo in order to identify and assign individuals into the appropriate number of clusters (K). Ten replicates were run for each value of K (ranging from K = 1 to K = 10) with a burn-in length of 200 000 and 1 000 000 MCMC iterations, using the admixture model and correlated allele frequencies settings. The resulting files were analysed in STRUCTURE HARVESTER [34] and the R script Structure-sum [35] to select the optimal value for K. Cluster assignments were further inspected using CLUMPAK [36] and visualized using distruct [37]. Due to the large number of shared alleles between the closely related taxa, and in order to rule out other potential parentages than the ones hypothesized for the two putative hybrids, we ran the STRUCTURE analysis on the total dataset rather than two separate analyses on the smaller datasets. Hybrid indices were estimated for all samples in both the *C*. *rostrata* var. *borealis* and *C*. *stenolepis* datasets using the INTROGRESS package in R [38, 39].

### Pollen staining analysis

Voucher specimens collected in the male flowering stage were examined to evaluate and compare pollen stainability (i.e., quality, as a measure of possible fertility) of the putative hybrids with their assumed parents. Investigation of pollen in *C*. *stenolepis* and its parents turned out to be impossible due to lack of available plants of *C*. *stenolepis* in male flowering stage. A total of 31 samples were included in the pollen quality analysis of *C*. *rostrata* (8 samples), *C*. *rostrata* var. *borealis* (15) and *C*. *rotundata* (8). Pollen was stained with a lactophenol-aniline blue solution (prepared according to [40]; described in [41]) and the stainability was recorded under a microscope 24 hours later. The pollen grains were classified as either well-stained (dark blue stain, indicating fertile pollen) or unstained and/or crumpled/deformed (indicating sterile pollen grains). With the exception of one sample where only 72 pollen grains could be found, approximately 200 pollen grains were investigated per sample. Finally, pollen stainability was given as the percentage of well-stained grains.

## Results

Of the 22 microsatellite loci tested, 15 were consistently amplified and scorable and thus applied in the further analyses (see Table 1). Out of 273 samples initially tested, a total of 231 samples were successfully amplified and genotyped for 12 or more of the markers. With the exception of four samples that each displayed three alleles for marker Carspe_4164c, and subsequently were removed from the dataset, only diploid genotype patterns were detected. In several cases, samples from the same population were genetically identical, and therefore believed to be from clonal shoots of the same individual. After removing all such redundant genotypes, the clone-corrected total dataset included 179 samples (69 *C*. *rostrata*, 19 *C*. *rostrata* var. *borealis*, 24 *C*. *rotundata*, 25 *C*. *vesicaria*, 14 *C*. *stenolepis*, 28 *C*. *saxatilis* (both subsp. *saxatilis* and subsp. *laxa*), meaning that the *C*. *rostrata* var. *borealis* dataset consisted of a total of 112 samples and the *C*. *stenolepis* dataset of a total of 67 samples.

**Table 1:** Characteristics of the 15 polymorphic microsatellite markers developed for species of *Carex* section *Vesicariae*.

Locus		Primer sequences 5'–3' ^a^	Repeat motif	Size range (bp)	No. of alleles	Multiplex group	Fluorophore
Carspe_0515c	F	TGGAACTTGTAGCCATCCCC	(GA)14	119–161	16	II	ATTO550
	R	TCTCCTAGCCAACTGTGCTG					
Carspe_0878c	F	GCTTAGAGCACCTTGATGTCG	(CT)12	85–129	20	V	FAM
	R	AGGACCTCAATAAGAAGTAACACC					
Carspe_0983c	F	TGCTGACTAGCATGGATCTGG	(CA)12	210–236	12	I	ATTO550
	R	GGTAACTCCAATACTGGCACC					
Carspe_1285c	F	TGGAAATTGTTATGGCAAGGC	(TC)13	152–192	13	IV	FAM
	R	AAAGGTTCTGCACAGGATGC					
Carspe_1657c	F	CGGGTTGTTCCATGATCTACTG	(TG)12	185–245	20	IV	HEX
	R	GCATGCCTTGTACCAGCAAC					
Carspe_2310c	F	AATATGATCGACAGGTGTGTTG	(TC)13	127–155	10	III	ATTO550
	R	TCGGTTTTCTGTATTTTTACTGCTG					
Carspe_4007c	F	GTGGATACCAAGTTGAGCCC	(AG)13	200–230	10	I	HEX
	R	TATCCAGCATGCATCAACGC					
Carspe_4164c	F	AGAGCCTGTTCACATGACCG	(CT)12	126–148	12	IV	HEX
	R	ACTTGTTGCAGTTCGCTACAG					
Carspe_4590c	F	TGATGAACGGTATAACACACAC	(AC)12	153–173	9	V	HEX
	R	ATTTTGACAATCCTTGAAAGTACAG					
Carspe_4899c	F	GAACTCGCTGCATTCTCACC	(CT)12	169–215	21	I	FAM
	R	ATCCTCTTTGCTTCAAGTTACC					
Carspe_4984c	F	TGCAAGAAGTCTCAGCATCC	(TC)13	134–176	19	II	FAM
	R	TCAGCCTCAGTGAAGAACGG					
Carspe_5770c	F	GCGCGTGCACAGAGATAAAG	(GA)12	173–225	20	IV	ATTO550
	R	GGTGCCCCTCAAGAAAATCC					
Carspe_6381cA	F	GGTTTAACTTGGGCCTCACC	(CT)12	140–184	20	III	HEX
	R	TTTGCTATCCCCTGAGAGCG					
Carspe_6867s	F	AGGAAAACATGTCTGTGGCG	(TG)14	93–155	23	II	HEX
	R	AGTGCATAAAGTCTAGGGTGC					
Carspe_7395s	F	TCCTCTACCTCTAGTTATGGGC	(TC)13	155–194	19	III	FAM
	R	GCATTTATGGAGTGGGCCTG					

^a^ Primer sequences and repeat motifs were provided by ecogenics GmbH after the initial screening of 12 individuals. Remaining information on size range and number of alleles (based on 179 individuals) as well as multiplex groups and fluorophores is related to the present study.

While amplification was successful for most markers, a substantial number of samples did not amplify for two of the microsatellite loci. For marker Carspe_2310c, no alleles were detected for 27 out of 179 samples: for *C*. *saxatilis* 21/28 (75%), for *C*. *rotundata* 5/24 (21%), and for *C*. *rostrata* var. *borealis* 1/19 (5%) samples showed no signs of amplification. For marker Carspe_6867s, 13 samples failed to amplify; all for *C*. *rotundata* (13/24 (54%)). The samples that did amplify for these loci had easily scorable allelic profiles, so while deciding to remove the two loci from the total dataset, we retained marker Carspe_2310c in the *C*. *rostrata* var. *borealis* dataset and marker Carspe_6867s in the *C*. *stenolepis* dataset. Number of alleles per microsatellite locus ranged from 9 to 23 (Table 1). Of the total number of 244 alleles, 177 alleles (73%) were shared between at least two taxa. The number of private alleles for each taxon was: *C*. *rostrata* 22 (17%), *C*. *rostrata* var. *borealis* 3 (3%), *C*. *rotundata* 6 (9%), *C*. *vesicaria* 23 (14%), *C*. *stenolepis* 3 (3%) and *C*. *saxatilis* 10 (11%).

**Fig 1:**
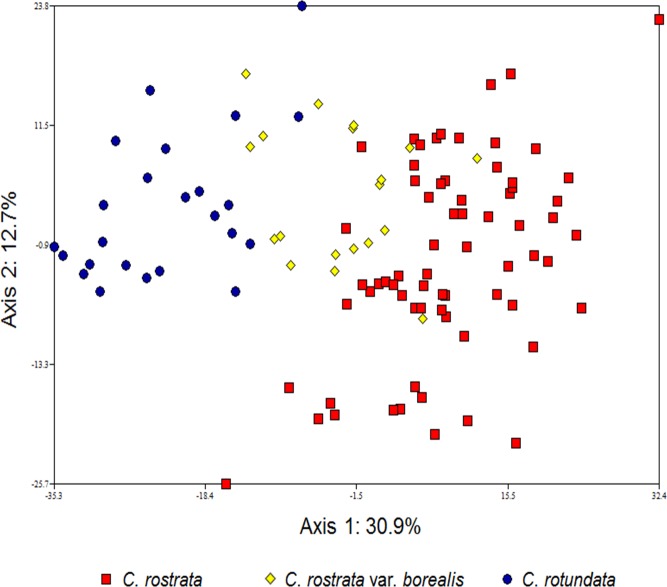
The first two axes of a principal components analysis (PCA) showing genetic differentiation between three taxa of *Carex* section *Vesicariae* (*C*. *rostrata*, *C*. *rostrata* var. *borealis* and *C*. *rotundata*) based on 14 microsatellite loci. Marker Carspe_6867s was excluded from this analysis as it did not amplify in all samples.

**Fig 2:**
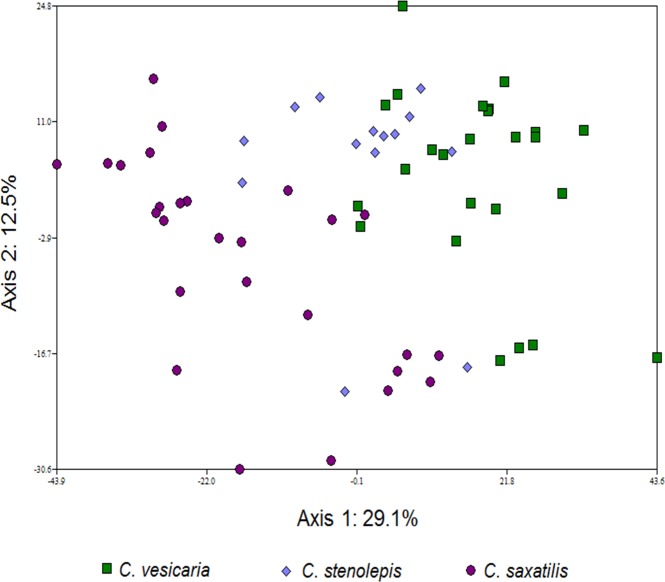
The first two axes of a principal components analysis (PCA) showing genetic differentiation between three taxa of *Carex* section *Vesicariae* (*C*. *vesicaria*, *C*. *stenolepis* and *C*. *saxatilis*) based on 14 microsatellite loci. Marker Carspe_2310c was excluded from this analysis as it did not amplify in all samples.

In the PCA analysis of the *C*. *rostrata* var. *borealis* dataset, *C*. *rostrata* and *C*. *rotundata* were quite well separated (no overlap between the two taxa) along the first principal component, accounting for 30.9% of the variation in the dataset (Fig 1). Samples of *C*. *rostrata* var. *borealis* appeared intermediate between *C*. *rostrata* and *C*. *rotundata*, with some overlap with each of the assumed parental species. The second principal component, accounting for 12.7% of the variation, failed to further distinguish the taxa. Similarly, in the PCA analysis of the *C*. *stenolepis* dataset, the *C*. *stenolepis* samples appeared intermediate between the hypothesized parental species along the first principal component, accounting for 29.1% of the variation in the dataset, with some samples overlapping with one or the other of the parents (Fig 2). In this analysis, samples from the two parental species had some overlap as well. The second principal component, accounting for 12.5% of the variation, failed to further distinguish the taxa.

**Fig 3:**
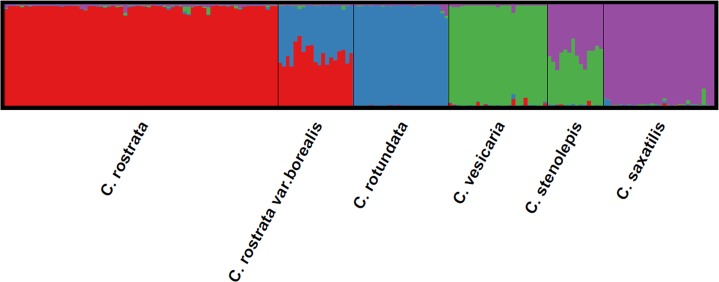
Graphical representation of the cluster assignment pattern for K = 4 based on STRUCTURE analysis of 13 microsatellite loci and 179 samples from six taxa in *Carex* section *Vesicariae*. Markers Carspe_6867s and Carspe_2310c were excluded from this analysis as they did not amplify in all samples.

From the results of the STRUCTURE analysis of the total dataset, we found that both K = 3 and K = 4 had acceptable similarity coefficients between runs (≈1.0) and unambiguous grouping of the taxa. However, the values for mean likelihood L (K) score and ΔK indicated that K = 4 was the optimal number of clusters. With this number of K, the clusters corresponded to the four parental taxa, *C*. *rostrata*, *C*. *rotundata*, *C*. *vesicaria* and *C*. *saxatilis* (Fig 3). The *C*. *rostrata* var. *borealis* samples were admixed, combining a roughly equal number of alleles from *C*. *rostrata* and *C*. *rotundata*. Similarly, the *C*. *stenolepis* samples combined roughly equal numbers of alleles from *C*. *vesicaria* and *C*. *saxatilis*. The tentative presence of two subspecies of *C*. *saxatilis* (subsp. *saxatilis* and subsp. *laxa*) was not reflected in this or any of the other analyses.

**Fig 4:**
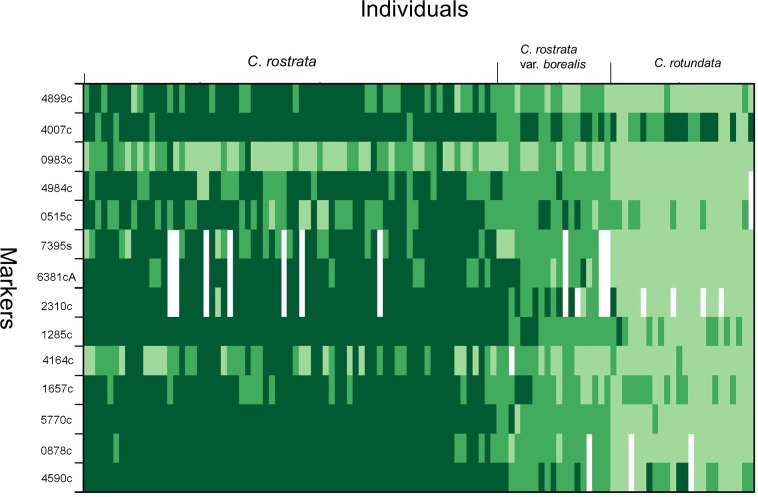
Plot of introgression patterns for 14 microsatellite loci and 112 samples in the *C*. *rostrata* var. *borealis* dataset including three taxa in *Carex* section *Vesicariae*: the hybrid *C*. *rostrata* var. *borealis* (19 samples) and its putative parental species *C*. *rostrata* (69 samples) and *C*. *rotundata* (24 samples). *Carex rostrata* is here defined as population 1 (P1), *C*. *rotundata* as population 2 (P2). Each rectangle in the plot represents an individual’s genotype at any given locus. The colours represent the ancestry of the genotype: dark green signifies a P1/P1 (*C*. *rostrata*) derived genotype, green a P1/P2 (mixed) genotype and light green a P2/P2 (*C*. *rotundata*) genotype. White rectangles indicate missing data. Marker Carspe_6867s was excluded from this analysis as it did not amplify in all samples.

**Fig 5:**
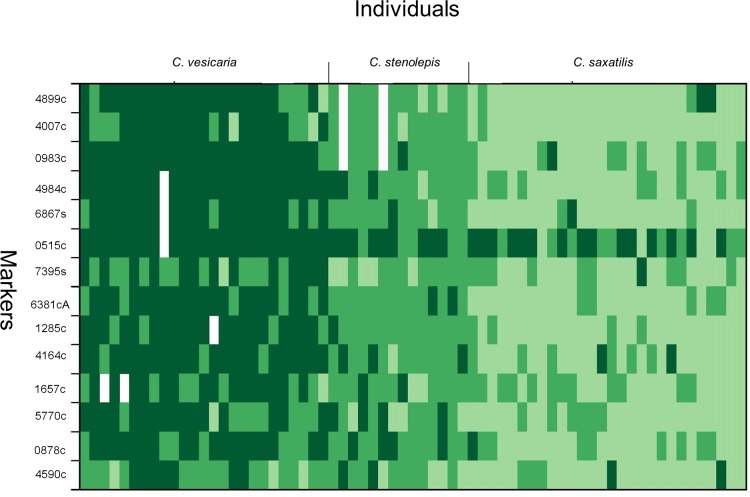
Plot of introgression patterns for 14 microsatellite loci and 67 samples in the *C*. *stenolepis* dataset including three taxa in *Carex* section *Vesicariae*: the hybrid *C*. *stenolepis* (14 samples) and its putative parents *C*. *vesicaria* (25 samples) and *C*. *saxatilis* (28 samples). *Carex vesicaria* is here defined as population 1 (P1), *C*. *saxatilis* as population 2 (P2). Each rectangle in the plot represents an individual’s genotype at any given locus. The colours represent the ancestry of the genotype: dark green signifies a P1/P1 (*C*. *vesicaria*) derived genotype, green a P1/P2 (mixed) genotype and light green a P2/P2 (*C*. *saxatilis*) genotype. White rectangles indicate missing data. Marker Carspe_2310c was excluded from this analysis as it did not amplify in all samples.

**Fig 6:**
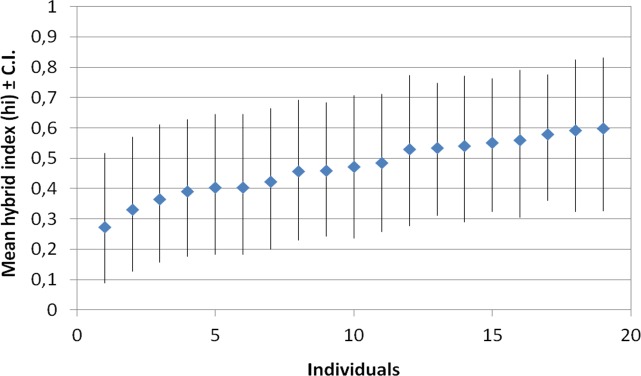
Estimates of hybrid index and associated lower and upper 95% confidence intervals for 19 *Carex rostrata* var. *borealis* samples based on 14 microsatellite loci. Marker Carspe_6867s was excluded from this analysis as it did not amplify in all samples.

**Fig 7:**
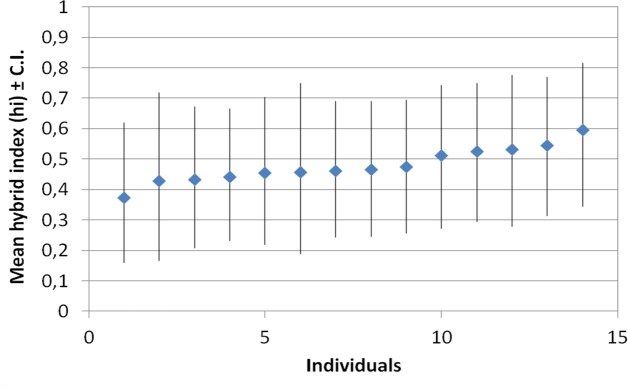
Estimates of hybrid index and associated lower and upper 95% confidence intervals for 14 *Carex stenolepis* samples based on 14 microsatellite loci. Marker Carspe_2310c was excluded from this analysis as it did not amplify in all samples.

The results from the INTROGRESS analyses indicated extensive interspecific heterozygosity and considerable variation in multilocus genotypes within samples from both of the putative hybrids *C*. *rostrata* var. *borealis* (Fig 4) and *C*. *stenolepis* (Fig 5). Estimates of mean hybrid index for *C*. *rostrata* var. *borealis* individuals ranged from 0.27 to 0.60 (Fig 6); the index ranged from 0 to 1, with 0 defined as entirely *C*. *rostrata* and 1 as entirely *C*. *rotundata*. Similarly, estimates of mean hybrid index for *C*. *stenolepis* individuals ranged from 0.37 to 0.60 (Fig 7); in this case, 0 was defined as entirely *C*. *vesicaria* and 1 as entirely *C*. *saxatilis*.

Pollen stainability was examined in *C*. *rostrata* var. *borealis* and its putative parental species *C*. *rostrata* and *C*. *rotundata* (Table 2). In *C*. *rostrata*, pollen stainability was high in all samples (93 – 99% stainability). In *C*. *rotundata* the numbers varied more, but these too were generally high (76 – 96%). The *C*. *rostrata* var. *borealis* samples had far lower percentages of stainable pollen compared to the parents. Stainability ranged from 3 to 37%: of the 15 samples, five had less than 10% stainable pollen, five had 10 – 19%, two had 20 – 29% and three had 30 – 37%.

**Table 2:** Measures of pollen stainability in *Carex rostrata*, *C*. *rostrata* var. *borealis* and *C*. *rotundata*.

Taxon and locality	Sample no.	Pollen stainability (%)
***Carex rostrata***		
Tromsø, Troms (NOR)	T063b/2	99
Tana, Finnmark (NOR)	T142/1	99
Tromsø, Troms (NOR)	T063b/1	96
Tynset, Hedmark (NOR)	T007/1	95
Båtsfjord, Finnmark (NOR)	T109/1	95
Båtsfjord, Finnmark (NOR)	T109/2	94
Tana, Finnmark (NOR)	T142/2	94
Tynset, Hedmark (NOR)	T007/2	93
***Carex rostrata* var. *borealis***		
Tromsø, Troms (NOR)	T050/1	37
Menesjavri, Inarin Lappi (FIN)	T099/1	31
Os, Hedmark (NOR)	T018/1	30
Tromsø, Troms (NOR)	T050/2	29
Os, Hedmark (NOR)	T018/2	23
Menesjavri, Inarin Lappi (FIN)	T099/2	18
Røros, Sør-Trøndelag (NOR)	T292/1	12
Storfjord, Troms (NOR)	T071/1	11
Kilpisjärvi, Enontekiön Lappi (FIN)	T076/1	11
Kilpisjärvi, Enontekiön Lappi (FIN)	T076/2	10
Båtsfjord, Finnmark (NOR)	T108/2	7
Kvalsund, Finnmark (NOR)	T162/1	6
Kvalsund, Finnmark (NOR)	T163/2	6
Kvalsund, Finnmark (NOR)	T162/2	5
Kvalsund, Finnmark (NOR)	T163/1	3
***Carex rotundata***		
Røros, Sør-Trøndelag (NOR)	T011a/2	96
Røros, Sør-Trøndelag (NOR)	T011a/1	93
Säytsjärvi, Inarin Lappi (FIN)	T104/4	93
Røros, Sør-Trøndelag (NOR)	T016/1	92
Røros, Sør-Trøndelag (NOR)	T016/5	87
Säytsjärvi, Inarin Lappi (FIN)	T104/1	84
Sør-Varanger, Finnmark (NOR)	T139/1	76
Sør-Varanger, Finnmark (NOR)	T139/5	76

## Discussion

### Hybrid origin of *Carex rostrata* var. *borealis* and *C*. *stenolepis*

The results of the STRUCTURE, PCA and INTROGRESS analyses all support the hypothesis that *C*. *rostrata* var. *borealis* and *C*. *stenolepis* represent interspecific hybrids between, respectively, *C*. *rostrata* and *C*. *rotundata*, and *C*. *vesicaria* and *C*. *saxatilis*. All four parental species were rather uniform in microsatellite patterns, including *C*. *saxatilis* (i.e., samples assigned in the field to subsp. *laxa* did not differ from samples of subsp. *saxatilis*). The pattern of allelic diversity seen in both *C*. *rostrata* var. *borealis* and *C*. *stenolepis* suggests that the hybrids have formed multiple times, as the multilocus genotypes observed varied considerably between individual samples in both taxa. If these hybrid lineages had been formed only once or a very few times, the genetic diversity observed among populations suggests that the hybridization events have taken place so long ago that the multilocus genotypes have been able to spread to their current wide geographic ranges and create the observed inter-population diversity.

Alternatively, a single postglacial hybridization event could also be invoked but would require considerable gene flow between the hybrids and/or their respective parental species, however, this scenario is unlikely given that clonal growth appears much more common than seed-set in populations of these taxa. Our opinion is that the rarity of private alleles found in *C*. *rostrata* var. *borealis* (3) and *C*. *stenolepis* (3), coupled with the observed high variation within each taxon, lends support to a model of multiple hybrid origins of both taxa (Figs 4 and 5). Furthermore, the extensive interspecific heterozygosity observed in individuals of *C*. *rostrata* var. *borealis* (mean hybrid indices ranging from 0.27 to 0.60; Fig 6) and *C*. *stenolepis* (mean hybrid indices ranging from 0.37 to 0.60; Fig 7) is consistent with hybrids of recent origin (see e.g., [42–44]). The genotypes of the hybrids indicate no predominant patterns of backcrossing towards any of the parents, but the rather few loci applied in our analysis make it difficult to draw conclusions about potential patterns at the genomic scale.

The chromosome numbers reported from the European species of section *Vesicariae* are in the range 2n = 60 – 88 [2, 16, 17]. A chromosome count for *C*. *stenolepis* reports 2n = ca. 80 [45]; quite similar to the reports for *C*. *vesicaria*: 2n = 70 – 88 (Reznicek and Ford [17] report 2n = 70, 74, 82, 88, Egorova [2] reports 74, 82, 86, 88, Elven et al. [16] report 70 – 88) and *C*. *saxatilis*: 2n = 78 – 80 ([17], whereas both [2] and [16] report only 2n = 80). For *C*. *rostrata* 2n = 60 – 82 (Reznicek and Ford [17] report only 2n = 60, Egorova [2] reports 60, 72 – 74, 76, 82, Elven et al. [16] report 60 – ca. 78), whereas for *C*. *rotundata* 2n = 80 [2, 16, 17].

Chromosomal fission and fusion is suggested as the dominant mode of karyotype evolution in *Carex* [46, 47] and this was confirmed for section *Vesicariae* by Lipnerová et al. [48]. Polyploidy, and particularly allopolyploidy, is considered to be rare in *Carex* and generally not responsible for the high chromosome numbers in the genus [46–52]. This, in addition to observations of only diploid microsatellite patterns in our samples (with the exceptions mentioned above), and the fact that *C*. *stenolepis* and *C*. *rostrata* var. *borealis* each are products of hybridization between two closely related species, makes it reasonable to propose that both *C*. *stenolepis* and *C*. *rostrata* var. *borealis* are homoploid hybrids, although to date, there are no reports of chromosome numbers available for the latter. Homoploid hybrid taxa and evidence for homoploid hybrid speciation is difficult to detect [53–56], but has previously been reported from section *Phacocystis* [11, 57], another ecologically important group of frequently hybridizing sedges found in arctic and boreal zones.

The microsatellite loci developed for this study proved to be very valuable and well suited not only in distinguishing the species in section *Vesicariae*, but also in detecting hybrids between them. In addition to resolving the origin of *C*. *rostrata* var. *borealis* and *C*. *stenolepis*, the markers were efficient in identifying other hybrids (assumed primary ones) among the *Vesicariae* species (see S2 Fig), hybrids not immediately recognized during field collection of the samples but confirmed on morphological evidence after the microsatellite analysis. The patterns of mixed ancestry seen in *C*. *rostrata* var. *borealis* and *C*. *stenolepis* in the STRUCTURE analysis were very similar to those of the additional hybrids (assumed primary) found in our study. The only difference is that the latter hybrids are rare and without any eco-geographical pattern, whereas *C*. *rostrata* var. *borealis* and *C*. *stenolepis* are frequent and ecological consistent elements in the more northern and upland Fennoscandian mires.

### Reproduction and clonality in *Carex rostrata* var. *borealis* and *C*. *stenolepis*

The low pollen stainability seen in *C*. *rostrata* var. *borealis* (i.e., mean 16%, n = 15) compared to the parental species *C*. *rostrata* and *C*. *rotundata* (means 96% and 87%, respectively, n = 8 for both species) suggests a limited capacity for the hybrid to engage in sexual reproduction. No samples of *C*. *rostrata* var. *borealis* showed a stainability level indicating that this taxon is nearly as fertile as its parental species. Unfortunately, we were unable to obtain any pollen data for *C*. *stenolepis* in our study, but a previous study by Ford et al. [28] measured pollen stainability in *C*. *stenolepis* and several other named taxa assumed to be hybrids of the same parentage. These authors found pollen stainability levels to be quite variable in both *C*. *stenolepis*, ranging from 0 to 83% (mean 36%, n = 13) and in *C*. *grahamii* Boott (a taxon regarded by these authors, and also by e.g., [24] and [26], to have the same parents as *C*. *stenolepis*), ranging from 0 to 36% (mean 7%, n = 7). Hylander [24] provides a thorough discussion of *C*. *stenolepis* and lists several reasons why he considered it an independent species: it often occurs in the absence of one or both of the putative parents *C*. *saxatilis* and *C*. *vesicaria*, has certain distinct morphological characters, shows little variation in morphology and has a fruit set comparable to that of the parental species. Other investigators (including us) have found differently, as we discuss below.

Even though some of the *C*. *stenolepis* samples included in the study of Ford et al. [28] had quite high pollen stainability, the majority had low values. These authors concluded that *C*. *stenolepis* (incl. *C*. *grahamii*) consists of largely sterile hybrids and should not be regarded as a “good” species (by this they probably mean a sexually reproducing species). Furthermore, considering the use of potential pollen stainability measures, they pointed out that “while this technique allows for the detection of obviously sterile pollen grains it cannot be considered a precise estimate of fertility since stained grains are not necessarily viable”, thereby regarding pollen stainability as a rough measure of pollen sterility rather than fertility. Ford et al. [28] found that some of the specimens of parental species displayed pollen stainability as low as 20%. They explained that this could be due to late sampling of specimens, when the anthers of the plants have mostly dehisced. We can add that at such a late stage nearly all well-developed grains are shed, whereas the undeveloped grains, of which there always is a certain percentage, are enriched in the samples studied.

While it is clear that individual plants of both *C*. *rostrata* var. *borealis* and *C*. *stenolepis* can exhibit moderately high levels of pollen stainability, this does not necessarily indicate that the taxa are able to reproduce sexually. Sexual reproduction depends, in the final instance, not on production of pollen grains but on production of seeds. According to P.W. Ball in [16], B.A. Ford examined a large number of specimens of *C*. *stenolepis* but was unable to find plants with well-formed and mature achenes. Hylander [24] also found *C*. *stenolepis* plants with seemingly empty perigynia, but argued that this was not necessarily a sign of hybridity, as failed fruit set could be due to external factors such as frost or flooding, and that fruit set frequently fails also in other species of section *Vesicariae*. An additional factor may be failing pollination due to weather conditions in the comparatively short period with male anthesis.

Although we agree that fruit set often can fail even in the four primary species (*C*. *rostrata*, *C*. *rotundata*, *C*. *saxatilis*, and *C*. *vesicaria*), the hybrid index results and the data on pollen stainability reported in both this study and the study of Ford et al. [28] give little support to suggestions that *C*. *rostrata* var. *borealis* and *C*. *stenolepis* are sexually reproducing species. It is therefore very unlikely that many if any of the stands have undergone significant evolutionary changes after the hybridization events. The very low number of private alleles in the two hybrid taxa compared to the high number of alleles shared with their respective parents indicates that the observed stands are based on single hybridization events, with little subsequent introgression with their parents and little or no stabilization by independent sexual processes. The STRUCTURE analysis clearly showed that both *C*. *rostrata* var. *borealis* and *C*. *stenolepis* combine approximately equal amounts of genetic material from their parents; again signifying recently formed hybrids.

The frequent and extended stands of both our hybrids are due to frequent co-occurrence of the parents, frequent hybridization, and extensive clonal growth of the hybrids. One explanation why *C*. *rostrata* var. *borealis* and *C*. *stenolepis* can be found even outside the ranges of their respective parents might lie in the longevity of these plants. Jónsdóttir et al. [58] estimated genet age in two clonal *Carex* species from section *Phacocystis* and found that whereas clones of *C*. *stans* Drejer (= *C*. *concolor* R. Br.) ranged from a rather modest 17 to 154 years old, the age of two clones of *C*. *ensifolia* Turcz. ex Ledeb. subsp. *arctisibirica* Jurtz. (= *C*. *bigelowii* Torr. subsp. *ensifolia* (Turcz. ex V. Krecz.), on the authority of [16]) was estimated to be well over 3000 years. Like these species, the species of section *Vesicariae* are largely clonal and able to form widespread and dense mats perhaps consisting of only a single genet (we commonly found genetic identity among samples from the same site in our study, often within distances of 50 – 100 m or more, and excluded such replicates). This is true also for the hybrids *C*. *rostrata* var. *borealis* and *C*. *stenolepis*.

If the predominant mode of reproduction in these plants is asexual (i.e., clonal by disruption of rhizome systems), their success does not rely much on production of viable pollen or successful fruit set. One might argue for the treatment of these taxa as hybrid species as they are widespread and form constant “populations” (i.e., stands) over a long period of time, certainly centuries and probably millennia. Given the long period of time involved, the parental species may have disappeared whereas the hybrids have remained, especially as the mires change during paludification and acidification. It is unlikely that competition plays any significant role. Co-occurrences, e.g., with *C*. *rostrata* var. *borealis* as an understorey in swards of *C*. *rostrata*, is a common situation, and *C*. *stenolepis* is rarely found within or close to stands of either *C*. *saxatilis* or *C*. *vesicaria*. However, it is important to note that the dispersal ability of such hybrids is very limited. The perhaps only possible diaspores are rhizome fragments, and the most likely agents of dispersal are water or birds.

### Taxonomic rank of *Carex rostrata* var. *borealis* and *C*. *stenolepis*

*Carex rostrata* var. *borealis* and *C*. *stenolepis* have been treated rather different taxonomically. Whereas the latter has been extensively discussed in numerous floras and other publications, the former has received almost no attention in the years since its description. *Carex rostrata* var. *borealis* has been, and still is, regarded merely as a variety of *C*. *rostrata* by the majority of authors, the exception being Elven [27] who proposes the current hybrid hypothesis. The discussion concerning *C*. *stenolepis* has rather focused on whether it should be accepted as a sexually reproducing species (e.g., [24, 29]) or an aggregate of recently formed, perhaps primary, hybrid (e.g., [28], see also [16]). As the two hybrid taxa in this study seem to display very similar patterns both with regards to microsatellite analyses and pollen stainability, we find it appropriate to treat them equally in taxonomic and nomenclatorial terms. The remaining question then is what taxonomic rank to assign to these problematic plants.

The results from this study affirm that both *C*. *rostrata* var. *borealis* and *C*. *stenolepis* are swarms of clonal hybrids that have been formed multiple times across various locations. Contrary to Hylander’s [24] claim that *C*. *stenolepis* plants are (at least) vegetatively very similar to one another, we found that the morphology of *C*. *stenolepis* varied quite a lot between locations, more so than the morphology of *C*. *rostrata* var. *borealis* did (field observations by A.T.M. Pedersen and R. Elven). Nevertheless, it is fairly easy to correctly identify both hybrids and to distinguish them from their parents. Also, *C*. *rostrata* var. *borealis* and *C*. *stenolepis* are much more common than other hybrids in section *Vesicariae*, and unlike these, they are significant constituents of the Fennoscandian mire vegetation, with regular and to a large degree predictable occurrence within consistent ranges, preferring habitats overlapping with, but not identical to, those of any of the four parents (*Carex rostrata* var. *borealis* in slightly more short-grown swards than typical of *C*. *rostrata* but often in company with *C*. *rotundata*; *C*. *stenolepis* in more swampy and shrubby mires than *C*. *saxatilis* (note the Norwegian name “vierstarr”, meaning “willow sedge”), but not in the swamps that *C*. *vesicaria* prefers).

Cayouette and Catling [59] wrote: “As with other groups of vascular plants, hybrids in sedges range from more or less fertile taxa that exist for long periods and dominate certain kinds of vegetation, to completely sterile taxa that occur rarely and only in rather unusual, ephemeral, disturbed situations in the presence of putative parents”. They furthermore stated that a binomial name would be useful for all hybrids that are common and well-documented. Following the arguments of these authors, and taking into consideration the above reasoning, we find it justified to accept them as hybrid species and thereby also accept binomial names for both taxa in this study. A new name for *C*. *rostrata* var. *borealis* together with a morphological review of this hybrid, and a reassessment of the nomenclature of what currently passes as *C*. *stenolepis*, will be presented elsewhere (Elven et al. in prep.).

## Supporting Information

S1 Fig Sampling maps for all specimens included in this study. (PDF)

S2 Fig Graphical representation of the cluster assignment pattern for K = 4 based on STRUCTURE analysis of 13 microsatellite loci and 179 samples from six taxa as well as 10 assumed primary hybrids in *Carex* section *Vesicariae*. Markers Carspe_6867s and Carspe_2310c were excluded from this analysis as they did not amplify in all samples.
(TIF)

S1 Table Collection information for all specimens included in this study. (XLSX)

## References

[1] Lid J, Lid DT. *Carex* L In Elven R, editor. Norsk flora. 7th ed. Oslo: Det Norske Samlaget; 2005 pp. 960–1022.

[2] Egorova TV. The sedges (*Carex* L.) of Russia and adjacent states (within the limits of the former USSR). St. Petersburg and St. Louis: St.-Petersburg State Chemical-Pharmaceutical Academy and Missouri Botanical Garden Press; 1999.

[3] Ball PW, Reznicek AA. *Carex* Linnaeus In Flora of North America Editorial Committee, editors. Flora of North America North of Mexico. Volume 23. Magnoliophyta: Commelinidae (in part): Cyperaceae. New York: Oxford University Press; 2002 pp. 254–572.

[4] Reznicek AA. Evolution in sedges (*Carex*, Cyperaceae). Can J Bot. 1990; 68(7): 1409–1432.

[5] Global *Carex* Group. Making *Carex* monophyletic (Cyperaceae, tribe Cariceae): a new broader circumscription. Bot J Linn Soc. 2015; 179(1): 1–42.

[6] Govaerts R, Simpson DA, Bruhl J, Egorova T, Goetghebeur P, Wilson K. World Checklist of Cyperaceae. Sedges. Royal Botanic Gardens, Kew: Kew Publishing; 2007.

[7] Starr JR, Naczi RFC, Chounaird BN. Plant DNA barcodes and species resolution in sedges (*Carex*, Cyperaceae). Mol Ecol Resour. 2009; 9(s1): 151–163. 21564974 10.1111/j.1755-0998.2009.02640.x

[8] Gebauer S, Starr JR, Hoffmann MH. Parallel and convergent diversification in two northern hemispheric species-rich *Carex* lineages (Cyperaceae). Org Divers Evol. 2014; 14(3): 247–258.

[9] Shekhovtsov SV, Shekhovtsova IN, Peltek SE. Phylogeny of Siberian species of *Carex* sect. *Vesicariae* based on nuclear and plastid markers. Nord J Bot. 2012; 30(3): 343–351.

[10] Cayouette J, Catling PM. Hybridization in the genus *Carex* with special reference to North America. Bot Rev. 1992; 58(4): 351–438.

[11] Korpelainen H, Virtanen V, Kostamo K, Väre H. Hybridization and introgression in *Carex aquatilis* and *C*. *paleacea*. Plant Syst Evol. 2010; 287(3): 141–151.

[12] Kukkonen I, Toivonen H. Taxonomy of wetland carices. Aquat Bot. 1988; 30(1–2): 5–22.

[13] Jiménez-Mejías P, Escudero M, Guerra-Cárdenas S, Lye KA, Luceño M. Taxonomic delimitation and drivers of speciation in the Ibero-North African *Carex* sect. *Phacocystis* river-shore group (Cyperaceae). Am J Bot. 2011; 98(11): 1855–1867. 10.3732/ajb.1100120 22025295

[14] The Global *Carex* Group. Megaphylogenetic specimen-level approaches to the *Carex* (Cyperaceae) phylogeny using ITS, ETS, and *matK* sequences: implications for classification. Syst Bot. 2016; 41(3): 500–518.

[15] Drury WH Jr. The ecology of the natural origin of a species of *Carex* by hybridization. Rhodora. 1956; 58(687): 51–72.

[16] Elven R, Murray DF, Razzhivin VY, Yurtsev BA. Annotated Checklist of the Panarctic Flora (PAF). Vascular Plants. Natural History Museum, University of Oslo 2011 Available: http://nhm2.uio.no/paf/

[17] Reznicek AA, Ford BA. *Carex* Linnaeus sect. *Vesicariae* In Flora of North America Editorial Committee, editors. Flora of North America North of Mexico. Volume 23. Magnoliophyta: Commelinidae (in part): Cyperaceae. New York: Oxford University Press; 2002 pp. 501–511.

[18] Ford BA, Ball PW, Ritland K. Allozyme diversity and genetic relationships among North American members of the short-beaked taxa of *Carex* sect. *Vesicariae* (Cyperaceae). Syst Bot. 1991; 16(1): 116–131.

[19] Ford BA, Ball PW. The taxonomy of the circumpolar short-beaked taxa of *Carex* sect. *Vesicariae* (Cyperaceae). Syst Bot. 1992; 17(4): 620–639.

[20] Schönswetter P, Elven R, Brochmann C. Trans-Atlantic dispersal and large-scale lack of genetic structure in the circumpolar, arctic-alpine sedge *Carex bigelowii* s. l. (Cyperaceae). Am J Bot. 2008; 95(8): 1006–1014. 10.3732/ajb.2007196 21632421

[21] Jermy C, Simpson DA, Foley MJY, Porter MS. Sedges of the British Isles. B.S.B.I Handbook No. 1. 3rd ed. London: Botanical Society of the British Isles; 2007.

[22] Blytt AG. Forsøg til en Theorie om Indvandringen af Norges Flora under vexlende regnfulde og tørre Tider. Nyt Magazin for Naturvidenskaberne. 1876; 21: 279–362.

[23] Stace CA, Preston CD, Pearman DA. Hybrid flora of the British Isles. Bristol: Botanical Society of Britain and Ireland; 2015.

[24] Hylander N. Nordisk kärlväxtflora – omfattande Sveriges, Norges, Danmarks, Östfennoskandias, Islands och Färöarnas kärlkryptogamer och fanerogamer. II. Stockholm: Almqvist et Wiksell; 1966.

[25] Bergeron A, Pellerin S. *Carex* x *cayouetti* (Cyperaceae), a new intersectional sedge hybrid from southern Québec, Canada. Phytoneuron. 2014; 52: 1–11.

[26] Laine U. Mikä on *Carex stenolepis*? (What is *Carex stenolepis*?) Memoranda Societatis pro Fauna et Flora Fennica. 1987; 63: 37–39.

[27] Elven R. *Carex rotundata* Wahlenb In Elven R, Fremstad E, Pedersen O, editors. Distribution maps of Norwegian vascular plants. IV. The eastern and northeastern elements. Trondheim: Akademika Publishing; 2013 pp. 139–141.

[28] Ford BA, Ball PW, Ritland K. Genetic and macromorphologic evidence bearing on the evolution of members of *Carex* section *Vesicariae* (Cyperaceae) and their natural hybrids. Can J Bot. 1993; 71(3): 486–500.

[29] Jakobsen A. En biosystematisk og autøkologisk studie over vierstarr (Carex stenolepis Less.). Cand. scient. Thesis, University of Trondheim. 1980.

[30] Holleley CE, Geerts PG. Multiplex Manager 1.0: a cross-platform computer program that plans and optimizes multiplex PCR. BioTechniques. 2009; 46(7): 511–517. 10.2144/000113156 19594450

[31] Kearse M, Moir R, Wilson A, Stones-Havas S, Cheung M, Sturrock S, et al Geneious Basic: an integrated and extendable desktop software platform for the organization and analysis of sequence data. Bioinformatics. 2012; 28(12): 1647–1649. 10.1093/bioinformatics/bts199 22543367 PMC3371832

[32] Rohlf F. NTSYSpc: Numerical taxonomy and multivariate analysis system. Version 2.11a New York: Exeter Software, Setauket, NY; 2000.

[33] Pritchard JK, Stephens M, Donnelly P. Inference of population structure using multilocus genotype data. Genetics. 2000; 155(2): 945–959. 10835412 10.1093/genetics/155.2.945 PMC1461096

[34] Earl DA, vonHoldt BM. STRUCTURE HARVESTER: a website and program for visualizing STRUCTURE output and implementing the Evanno method. Conserv Genet Resour. 2012; 4(2): 359–361.

[35] Ehrich D, Gaudeul M, Assefa A, Koch MA, Mummenhoff K, Nemomissa S, et al Genetic consequences of Pleistocene range shifts: contrast between the Arctic, the Alps and the East African mountains. Mol Ecol. 2007; 16(12): 2542–2559. 10.1111/j.1365-294X.2007.03299.x 17561912

[36] Kopelman NM, Mayzel J, Jakobsson M, Rosenberg NA, Mayrose I. CLUMPAK: a program for identifying clustering modes and packaging population structure inferences across *K*. Mol Ecol Resour. 2015; 15(5): 1179–1191. 10.1111/1755-0998.12387 25684545 PMC4534335

[37] Rosenberg NA. DISTRUCT: a program for the graphical display of population structure. Mol Ecol Notes. 2004; 4(1): 137–138.

[38] Buerkle CA. Maximum-likelihood estimation of a hybrid index based on molecular markers. Mol Ecol Notes. 2005; 5(3): 684–687.

[39] Gompert Z, Buerkle CA. INTROGRESS: a software package for mapping components of isolation in hybrids. Mol Ecol Resour. 2010; 10(2): 378–384. 10.1111/j.1755-0998.2009.02733.x 21565033

[40] Maneval WE. Lacto-phenol preparations. Stain Technol. 1936; 11(1): 9–11.

[41] Kearns CA, Inouye DW. Techniques for Pollination Biologists. Niwot: University Press of Colorado; 1993.

[42] Lamont BB, He T, Enright NJ, Krauss SL, Miller BP. Anthropogenic disturbance promotes hybridization between *Banksia* species by altering their biology. J Evol Biol. 2003; 16(4): 551–557. 14632219 10.1046/j.1420-9101.2003.00548.x

[43] Rentsch JD, Leebens-Mack J. Homoploid hybrid origin of *Yucca gloriosa*: intersectional hybrid speciation in Yucca (Agavoideae, Asparagaceae). Ecol Evol. 2012; 2(9): 2213–2222. 10.1002/ece3.328 23139880 PMC3488672

[44] Johnson MG, Lang K, Manos P, Golet GH, Schierenbeck KA. Evidence for genetic erosion of a California native tree, *Platanus racemosa*, via recent, ongoing introgressive hybridization with an introduced ornamental species. Conserv Genet. 2016; 17(3): 593–602.

[45] Borgen L, Elven R. Chromosome numbers of flowering plants from northern Norway and Svalbard. Nord J Bot. 1983; 3(3): 301–306.

[46] Hipp AL. Nonuniform processes of chromosome evolution in sedges (*Carex*: Cyperaceae). Evolution. 2007; 61(9): 2175–2194. 10.1111/j.1558-5646.2007.00183.x 17767589

[47] Hipp AL, Rothrock PE, Roalson EH. The evolution of chromosome arrangements in Carex (Cyperaceae). Bot Rev. 2009; 75(1): 96–109.

[48] Lipnerová I, Bureš P, Horová L, Šmarda P. Evolution of genome size in *Carex* (Cyperaceae) in relation to chromosome number and genomic base composition. Ann Bot. 2013; 111(1): 79–94. 10.1093/aob/mcs239 23175591 PMC3523652

[49] Heilborn O. Aneuploidy and polyploidy in *Carex*. Svensk Botanisk Tidskrift. 1932; 26: 137–146.

[50] Heilborn O. On the origin and preservation of polyploidy. Hereditas. 1934; 19(1–2): 233–242.

[51] Hipp AL, Reznicek AA, Rothrock PE, Weber JA. Phylogeny and classification of *Carex* section *Ovales* (Cyperaceae). Int J Plant Sci. 2006; 167(5): 1029–1048.

[52] Hipp AL, Rothrock PE, Reznicek AA, Berry PE. Changes in chromosome number associated with speciation in sedges: A phylogenetic study in *Carex* section *Ovales* (Cyperaceae) using AFLP data. Aliso. 2007; 23(1): 193–203.

[53] Mallet J. Hybrid speciation. Nature. 2007; 446: 279–283. 10.1038/nature05706 17361174

[54] von Hagen KB, Seidler G, Welk E. New evidence for a postglacial homoploid hybrid origin of the widespread Central European *Scabiosa columbaria* L. s. str. (Dipsacaceae). Plant Syst Evol. 2008; 274(3): 179–191.

[55] Clay DL, Novak SJ, Serpe MD, Tank DC, Smith JF. Homoploid hybrid speciation in a rare endemic *Castilleja* from Idaho (*Castilleja christii*, Orobanchaceae). Am J Bot. 2012; 99(12): 1976–1990. 10.3732/ajb.1200326 23211568

[56] Schumer M, Rosenthal GG, Andolfatto P. How common is homoploid hybrid speciation? Evolution. 2014; 68(6): 1553–1560. 10.1111/evo.12399 24620775

[57] Dragon JA, Barrington DS. Systematics of the *Carex aquatilis* and *C*. *lenticularis* lineages: Geographically and ecologically divergent sister clades of *Carex* section *Phacocystis* (Cyperaceae). Am J Bot. 2009; 96(10): 1896–1906. 10.3732/ajb.0800404 21622311

[58] Jónsdóttir IS, Augner M, Fagerström T, Persson H, Stenström A. Genet age in marginal populations of two clonal *Carex* species in the Siberian Arctic. Ecography. 2000; 23(4): 402–412.

[59] Cayouette J, Catling PM. Naming, filing, and conservation of *Carex* hybrids. Cyperaceae Newsletter. 1991; 9: 6–7.

