# Correction: Hybrid Origins of *Carex rostrata* var. *borealis* and *C*. *stenolepis*, Two Problematic Taxa in *Carex* Section *Vesicariae* (Cyperaceae)

**DOI:** 10.1371/journal.pone.0171398

**Published:** 2017-02-03

**Authors:** A. Tiril M. Pedersen, Michael D. Nowak, Anne K. Brysting, Reidar Elven, Charlotte S. Bjorå

The first author’s surname is incorrectly preceded by an initial. The correct citation is: Pedersen ATM, Nowak MD, Brysting AK, Elven R, Bjorå CS (2016) Hybrid Origins of *Carex rostrata* var. *borealis* and *C. stenolepis*, Two Problematic Taxa in *Carex* Section *Vesicariae* (Cyperaceae). PLoS ONE 11(10): e0165430. doi:10.1371/journal.pone.0165430.
