# Supplementary material for: Hybrid Origins of Carex rostrata var. borealis and C. stenolepis, Two Problematic Taxa in Carex Section Vesicariae (Cyperaceae)
Source: PLoS One. 2016 Oct 25;11(10):e0165430. doi: 10.1371/journal.pone.0165430 (PMC5079627; doi:10.1371/journal.pone.0165430)
Supplement: S1 Fig — (PDF) [file pone.0165430.s001.pdf]

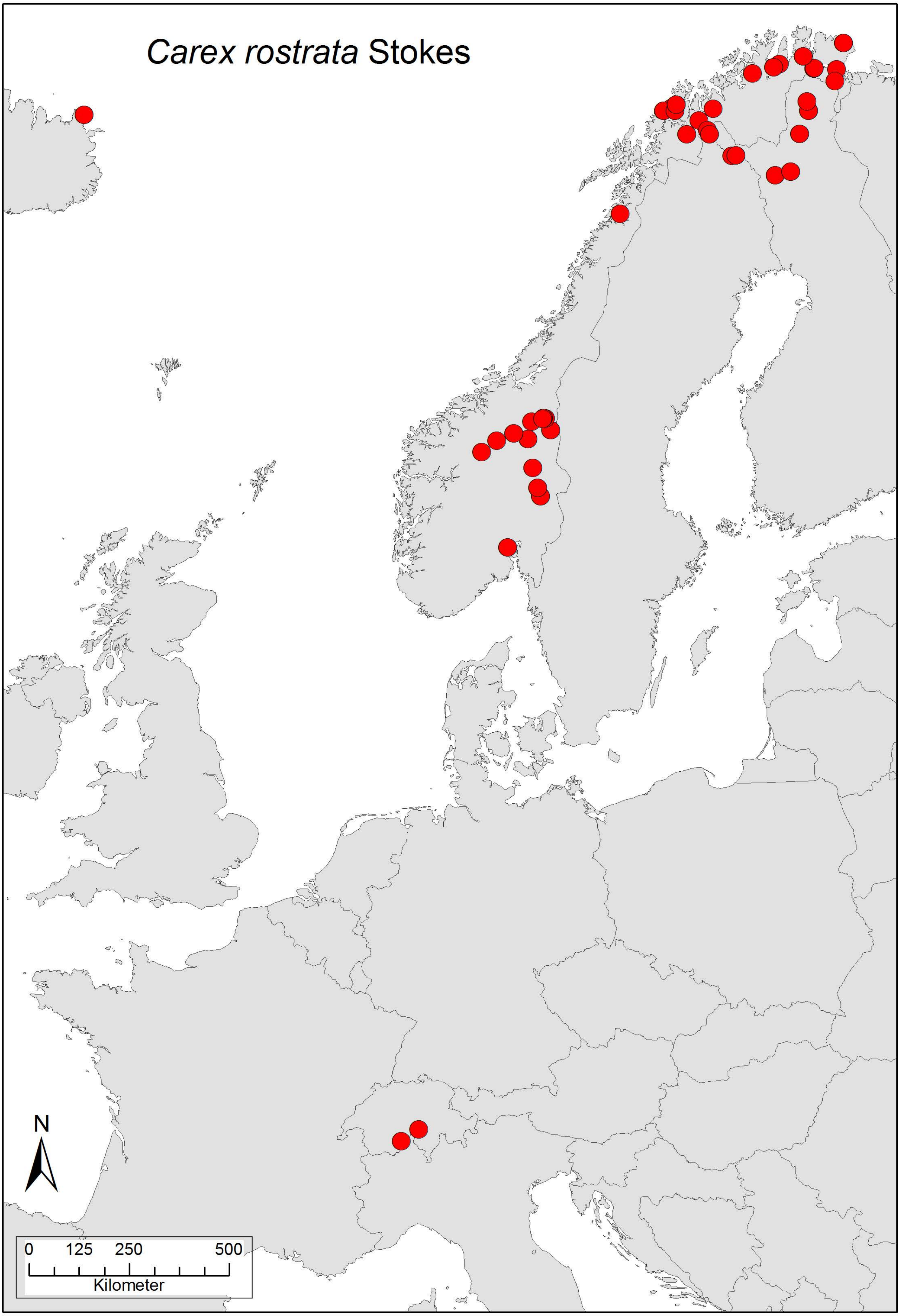
Carex rostrata Stokes
N
0 125 250 500
Kilometer

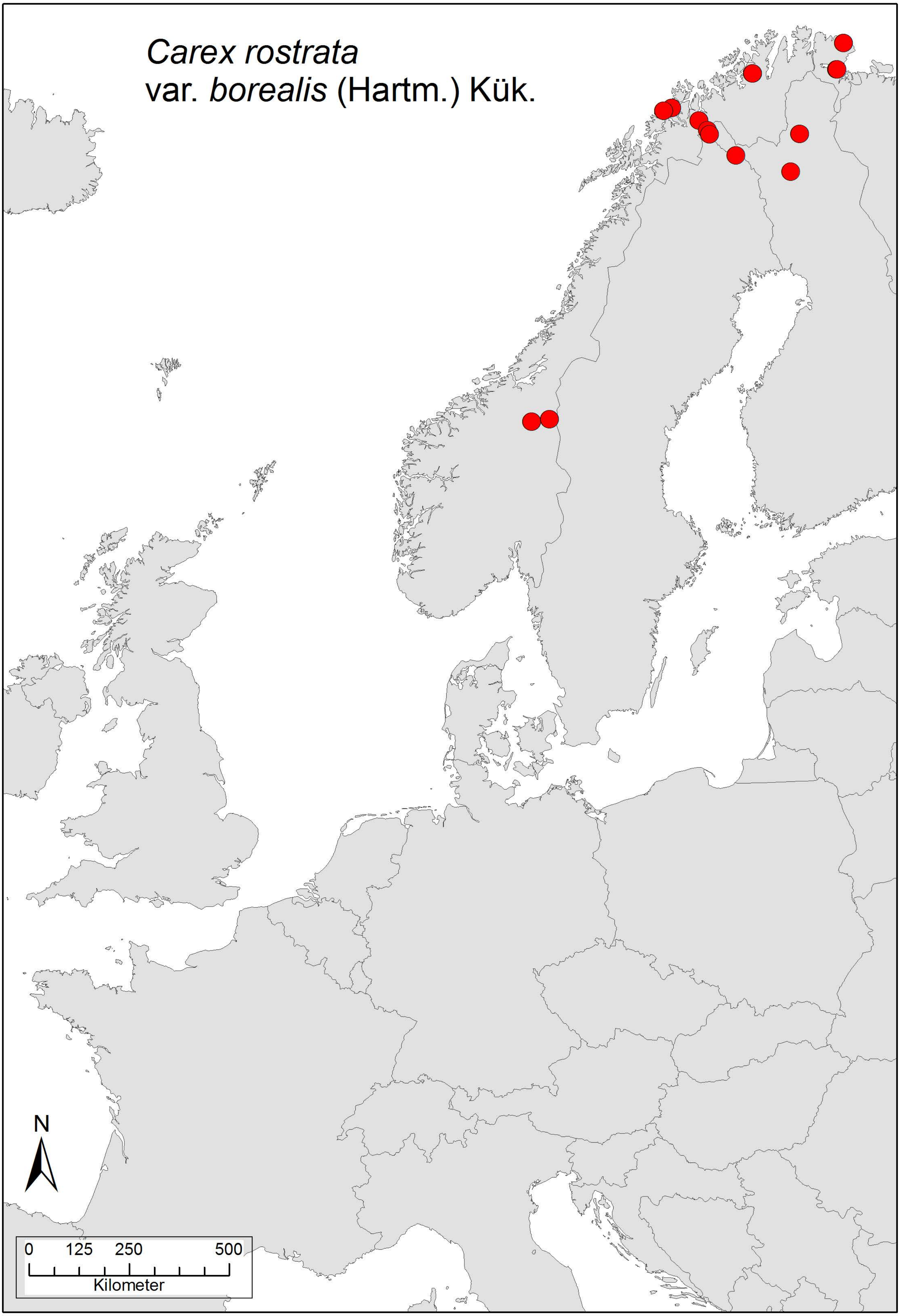
Carex rostrata
var. *borealis* (Hartm.) Kük.
N
0 125 250 500
Kilometer

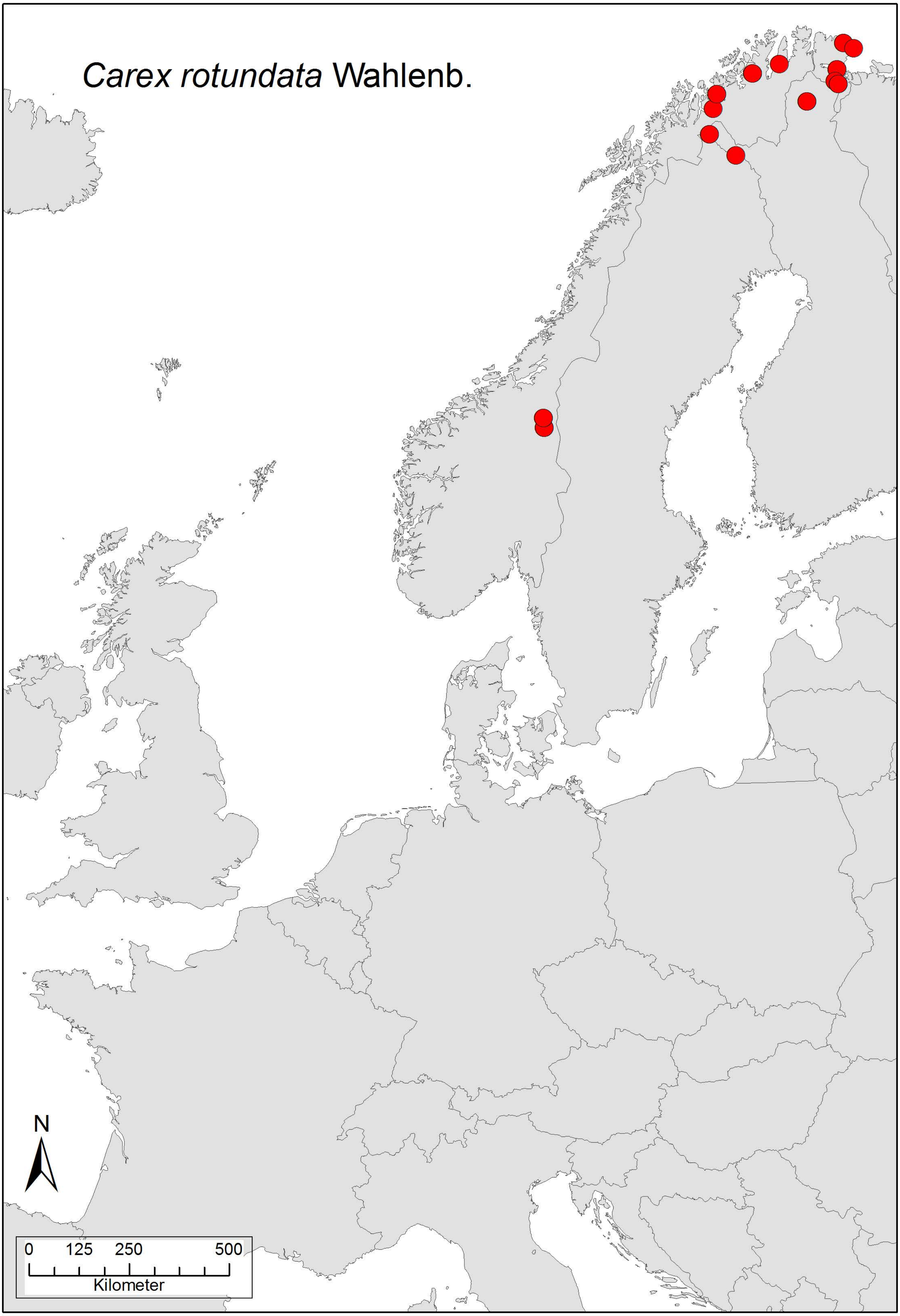
Carex rotundata Wahlenb.
N
0 125 250 500
Kilometer

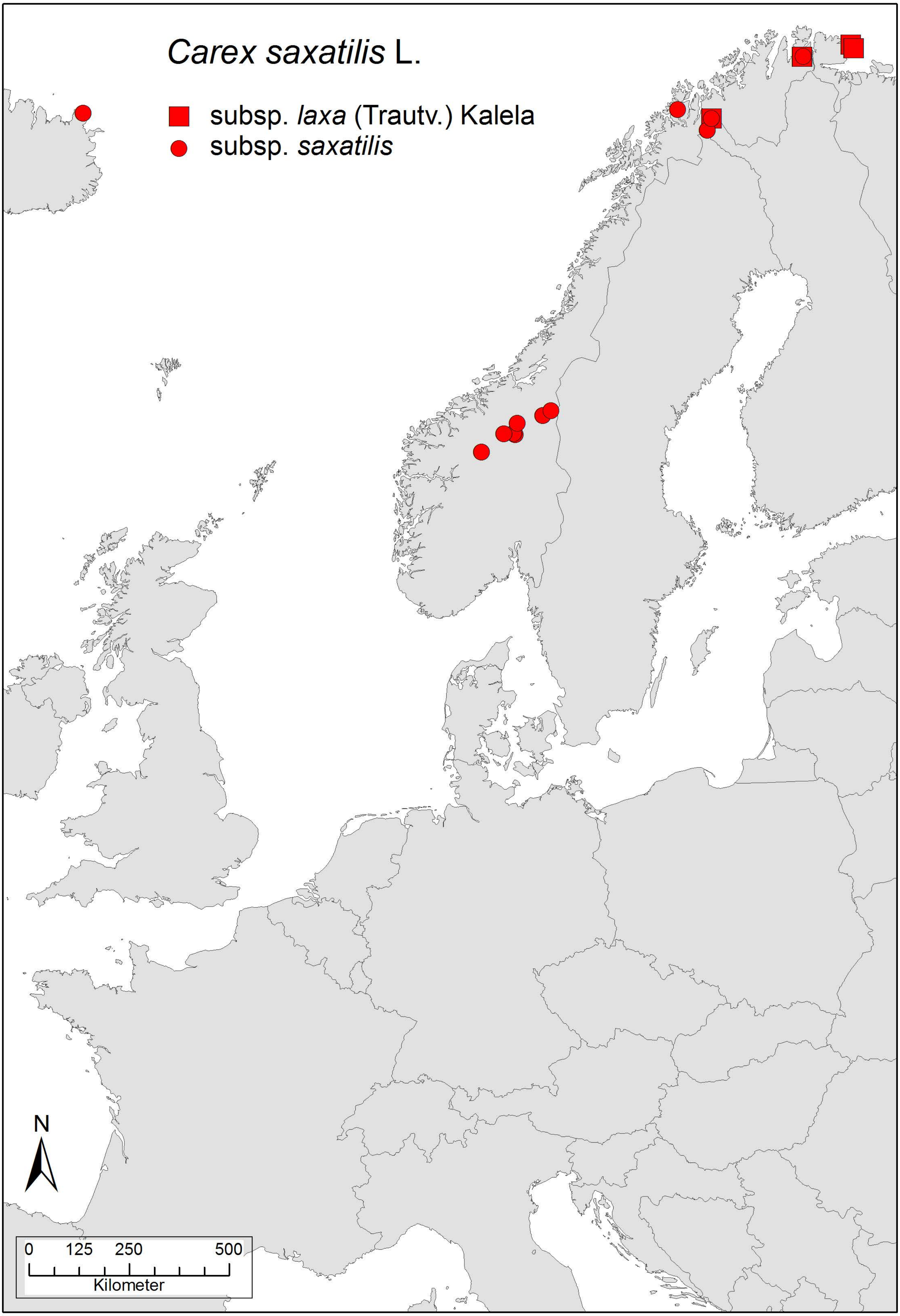

Carex saxatilis L.
subsp. *laxa* (Trautv.) Kalela
subsp. *saxatilis*
N
0 125 250 500
Kilometer

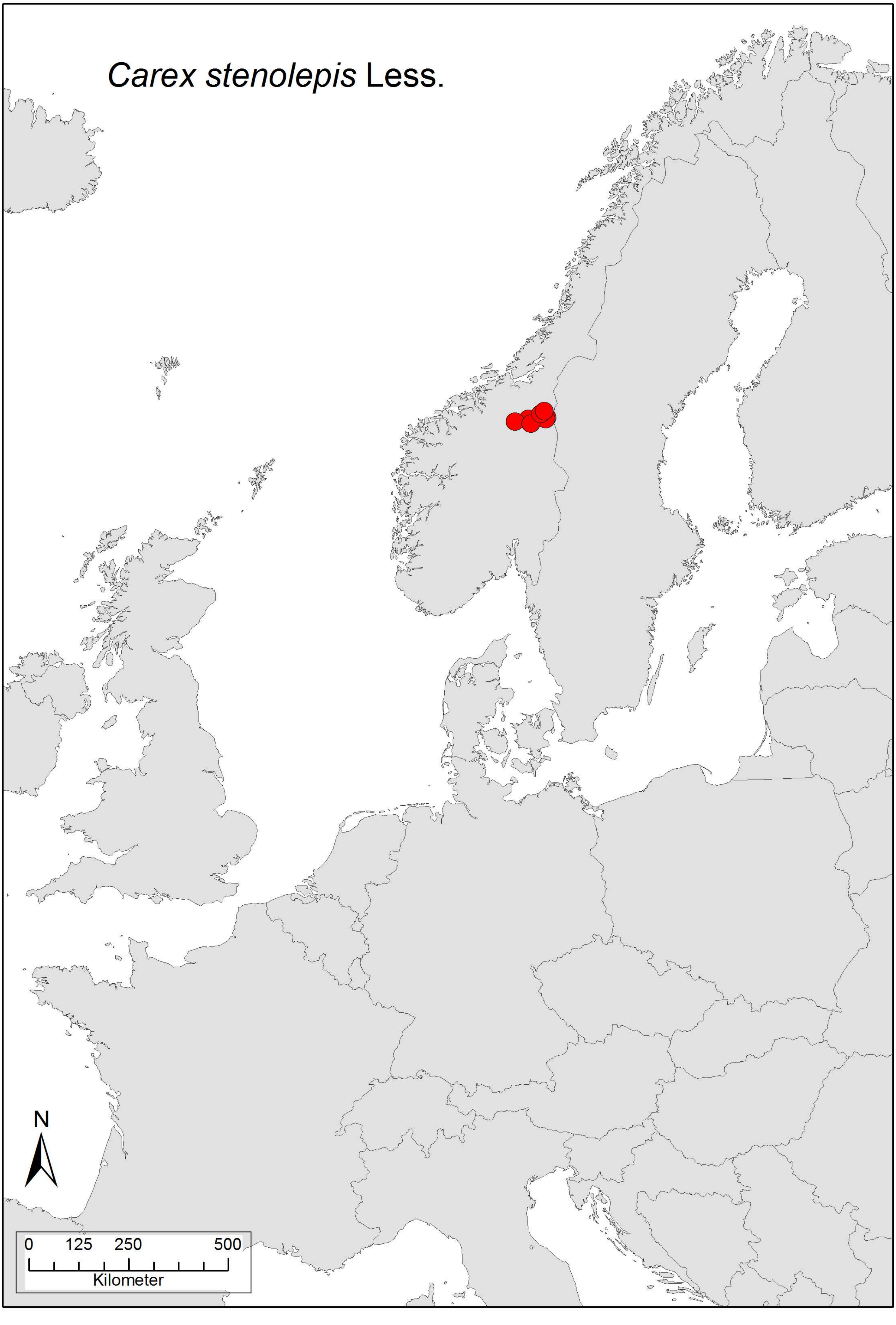
Carex stenolepis Less.
N
0 125 250 500
Kilometer

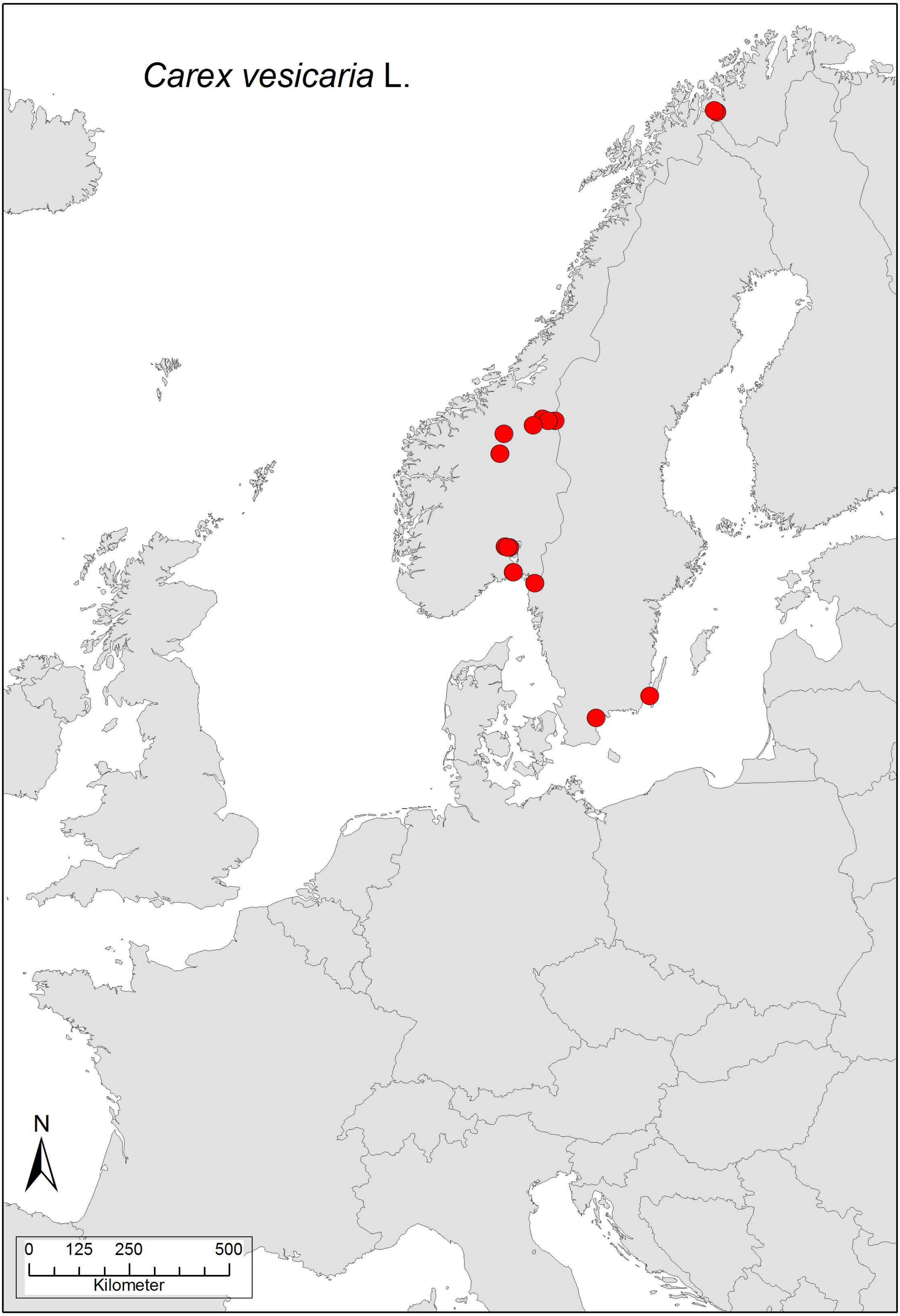
Carex vesicaria L.
N
0 125 250 500
Kilometer
