# Supplementary figures and images for: Hybrid Origins of Carex rostrata var. borealis and C. stenolepis, Two Problematic Taxa in Carex Section Vesicariae (Cyperaceae)
Source: PLoS One. 2016 Oct 25;11(10):e0165430. doi: 10.1371/journal.pone.0165430 (PMC5079627; doi:10.1371/journal.pone.0165430)

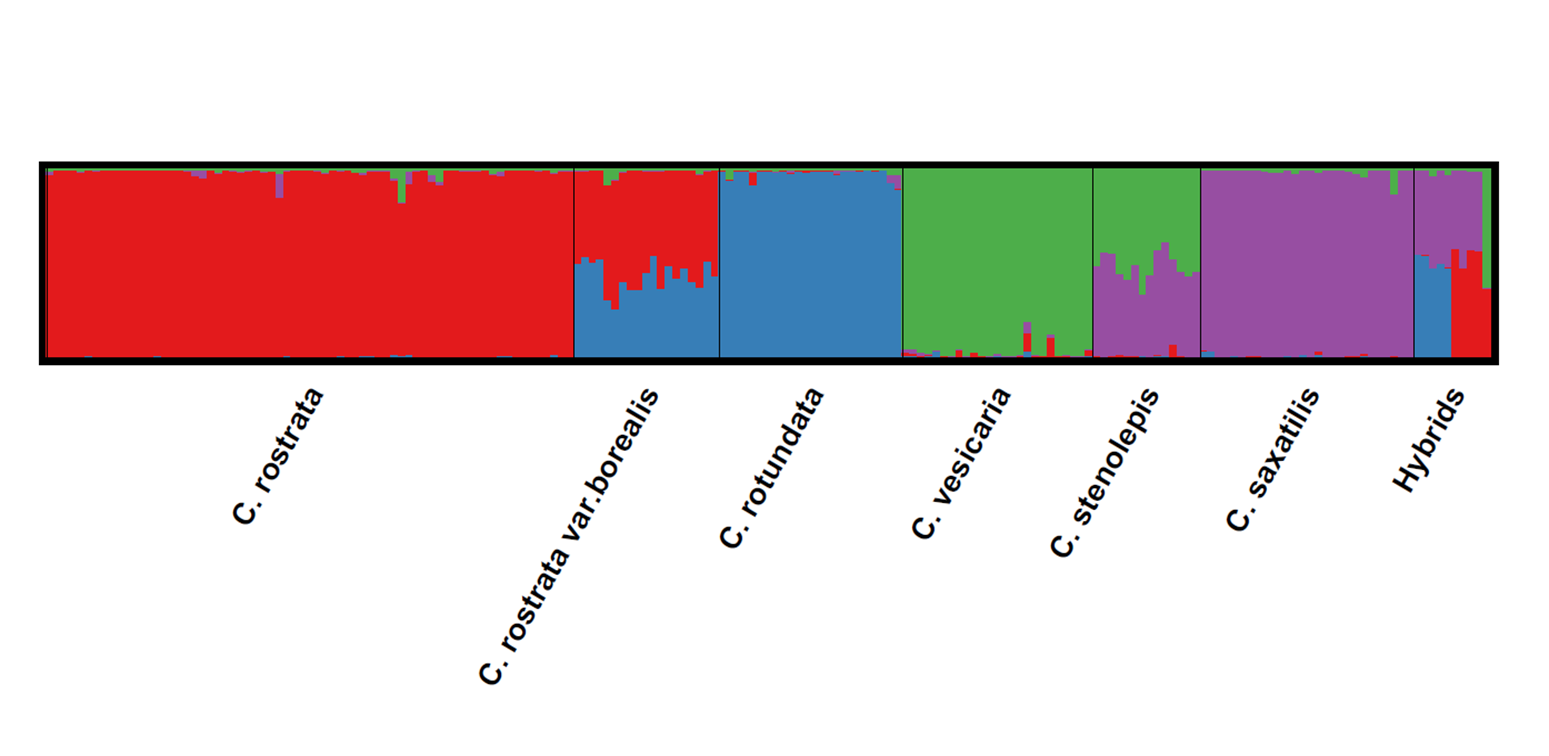

Supplement: S2 Fig — Markers Carspe_6867s and Carspe_2310c were excluded from this analysis as they did not amplify in all samples. (TIF) [file pone.0165430.s002.tif]
